# Molecular Modeling of N-Acetylglucosamine Binding to the I154R Mutant of NAGLU: Pathogenic Insights into Sanfilippo Syndrome Type B

**DOI:** 10.3390/ijms27104404

**Published:** 2026-05-15

**Authors:** Priyanka Kannan, Madhana Priya Nanda Kumar, Sidharth Kumar Nanda Kumar, Vasundra Vasudevan, Kuppan Kaviarasan, Magesh Ramasamy

**Affiliations:** 1 Department of Biotechnology, Sri Ramachandra Institute of Higher Education and Research (DU), Porur, Chennai 600116, India; priyankak@sriramachandra.edu.in (P.K.); madhanapriya7@gmail.com (M.P.N.K.); sidharthnandakumar01@gmail.com (S.K.N.K.); vasundravasudev@gmail.com (V.V.); 2 Department of Biomedical Sciences, Sri Ramachandra Institute of Higher Education and Research (DU), Porur, Chennai 600116, India

**Keywords:** mucopolysaccharidosis IIIB, NAGLU, health care, molecular docking, moleculardynamics simulation, MM/PBSA

## Abstract

Sanfilippo syndrome type B, also known as mucopolysaccharidosis type IIIB (MPS IIIB), is a rare autosomal recessive lysosomal storage disorder caused by mutations in the N-acetyl-α-D-glucosaminidase (*NAGLU*) gene, which encodes the enzyme α-N-acetylglucosaminidase. This enzyme is essential for degrading heparan sulfate. The deficiency leads to toxic accumulation within cells. To investigate the impact of NAGLU mutations, mutational data were retrieved from public databases including NCBI, UniProt, and HGMD. A total of 162 variants were evaluated using sequence-based prediction tools to identify deleterious mutations, followed by structure-based in silico analyses to assess changes in protein stability, biophysical properties, and ligand-binding potential. Among the analyzed mutations, the I154R variant was identified as the most deleterious, showing disease-associated characteristics, structural instability, and impaired functional properties. Molecular docking with N-acetylglucosamine (NAG) revealed binding affinities of −4.17 kcal/mol for the native protein and −3.97 kcal/mol for the I154R mutant, suggesting a retained yet slightly reduced binding potential. Molecular dynamics simulations supported these findings, indicating stable trajectories, favorable interaction profiles, and moderate flexibility for both complexes. These results enhance our understanding of NAGLU-related pathogenicity in MPS IIIB, contributing to improved health care strategies and offering a valuable foundation for future therapeutic developments targeting enzyme dysfunction in Sanfilippo syndrome type B.

## 1. Introduction

The lysosomal enzyme N-acetyl-glucosaminidase, which is responsible for the breakdown of the complex polysaccharide heparan sulfate, is encoded by mutations in the N-acetyl-α-D-glucosaminidase (*NAGLU*) gene, causing mucopolysaccharidosis IIIB (MPS IIIB) and an autosomal recessive metabolic condition. Advanced perceptual decline, behavioral issues and motor function impairment are characteristics of the condition [1,2]. Mucopolysaccharidosis type III (MPS III), also known as Sanfilippo syndrome, is a lysosomal storage disorder characterized by impaired degradation of heparan sulfate. It is subdivided into four types (A–D), each caused by deficiency of a distinct enzyme involved in the heparan sulfate degradation pathway. Among these, MPS IIIB is specifically associated with mutations in the *NAGLU* gene, which encodes the lysosomal enzyme α-N-acetylglucosaminidase. Deficiency of *NAGLU* results in the accumulation of partially degraded heparan sulfate within lysosomes, leading to the progressive neurodegeneration, behavioral abnormalities, and clinical heterogeneity observed in MPS IIIB patients. A lack of the proper lysosomal enzyme results in the inability to break down one or more glycosaminoglycans, which is the biochemical hallmark of the mucopolysaccharidosis family of lysosomal illnesses. Subtype B (MPS IIIB) results from mutations in *NAGLU*, the focus of this study [3]. Mucopolysaccharidosis are lysosomal disorders characterized by the inability to degrade glycosaminoglycans due to deficient lysosomal enzymes. In Sanfilippo syndrome (MPS III), the degradation of heparan sulfate is specifically impaired [4]. While the clinical course of MPS III can be separated into three phases, MPS IIIB exhibits clinical heterogeneity. After a first normal prenatal and early postnatal period, delayed cognitive development is evident in the first phase, which typically begins between the ages of 1 and 4. The second stage of the illness typically starts between three and five years of age and lasts between five and ten years [5].

In addition to learning challenges, significant behavioral disorders, hyperactivity, hostility that is extremely difficult to treat, sleep disturbances, sensor neural deafness, hernia, gastrointestinal complaints, and joint contracture [6], those with MPS type-III have a severe intellectual handicap. Six exons make up *NAGLU* cDNA, which codes for a 743 amino acid polypeptide with six asparagine-linked glycosylation sites and a cleavable signal sequence. The mature protein has a molecular mass of about 80 kDa based on its 720 amino acids [7]. Despite being a potentially fatal illness, MPS III is categorized as an uncommon disease with an incidence of 1 in 70,000 births [8]. Heparan sulfate’s s-linked N-acetyl glucosamine residues are hydrolyzed by acetylglucosaminidase. These moieties are either generated by acetyl-CoA during the lysosomal breakdown of GAG or are present naturally: oL-N-Acetylglucosaminidase and oL-glucosaminide transferase have been isolated from various human tissues. It has all of the typical characteristics of lysosomal glycosidase [9]. The most frequent mutations found in the study were missense mutations. A small number of individuals, mostly siblings, had mutations that resembled those of patients exhibiting relevant clinical traits. For certain NAGLU mutations, it has been suggested that there might be a phenotype–genotype association [10]. As there are 0.28–4.1 cases of MPS combined for every 100,000 live births; MPS III is the most prevalent kind. The most prevalent types, MPS III A and B, are responsible for 60% and 30% of Sanfilippo syndromes, respectively [11]. Geographical location affects prevalence, and some subtypes seem to be more common in particular parts of the globe. In general, varieties C and D are diagnosed less frequently than MPS IIIA and B [12].

A unique homotrimeric configuration is shown by the crystal structure, which is mainly preserved by hydrophobic and electrostatic contacts through domain II of three adjacent domains from the N-to C-terminus [13]. As of right now, palliative care and symptom management are the only options for treating MPS III patients. Treatments specific to MPS III are being investigated, such as gene therapy, hematopoietic stem cell transplantation, intrathecally administered enzyme replacement therapy, and substrate reduction therapy [14].Emerging therapeutic approaches targeting NAGLU deficiency in MPS IIIB include enzyme replacement strategies using recombinant human NAGLU (rhNAGLU), which have been explored to restore lysosomal enzyme activity and facilitate degradation of accumulated heparan sulfate. More advanced approaches include modified forms of NAGLU such as Tralesinidase alfa, a fusion protein engineered to enhance cellular uptake and improve central nervous system (CNS) delivery. This modification utilizes receptor-mediated pathways to overcome limitations associated with the blood–brain barrier. Additionally, NAGLU-IGF2 fusion constructs represent next-generation therapeutic strategies aimed at improving lysosomal targeting and enzymatic efficiency. Cognitive and neurological impairments are primary clinical features of MPS III. Addressing these involves supportive care and targeted management of associated complications, with treatment complexities stemming from the presence of the blood–brain barrier (BBB) [15].Computational analyses were performed to investigate genotype–phenotype correlations. Based on the above results, I154R and W649C were subjected to the HOPE project analysis, which revealed that I154R mutants had a loss of hydrophobic interactions in the core protein, mutational screening to understand the protein sequence and structures was carried out by incorporating various algorithms with the help of single nucleotide polymorphisms with Mucopolysaccharidosis subgroups like MPS I, IIIA and VI [16,17,18,19].

In this study, the mutagenic properties were evaluated using various computational tools and software to predict the functionally significant mutant proteins that are responsible for MPS IIIB. ConSurf was utilized to investigate and gather information regarding evolutionary changes in amino acid substitutions among the protein sequences and its conserved region [20,21]. The mutational properties were examined to identify the most vital amino acid mutants using various in silicostudies on the NAGLU protein. The methods that follow sequence-based screening are pathogenicity identification (Meta-SNP (SNAP, MAPP, PANTHER, PhD-SNP, SIFT), SNPs&GO and PredictSNP prediction), investigation of the biophysical properties, stability analysis, SNPeffect, and HOPE. The structure-based screening methodology was performed with the most significant mutant protein structures, which were identified based on the sequence-based analysis carried out by the interaction profiling and interatomic simulation study to determine the effects of the structure causing protein assembly [22,23,24].

## 2. Results

### 2.1. Investigating the Missense Dataset and Evolutionary Patterns

The dataset, comprising 162 missense mutations, was sourced from ClinVar (NCBI), HGMD, and UniProt for the NAGLU protein (Table 1). Variants reported in multiple databases were identified and consolidated during dataset compilation. Such variants are represented as combined entries with multiple database identifiers. Therefore, the final dataset consists exclusively of unique (non-redundant) nsSNPs, and no duplicate variants were retained (Supplementary Table S1). Following this, a thorough examination of insilico pipelines was conducted to identify mutations with promising attributes. Subsequently, the retrieved mutations underwent evolutionary analysis using the NAGLU protein in FASTA format, which was obtained from the UniProt database. The output that was produced gave a scale range for each amino acid residue from 1 to 9 (Supplementary Table S1). Notably, 45 mutations attained a score value of 9, signifying their classification within a highly conserved domain (Table 2).

### 2.2. An Investigation of Pathogenicity and Biophysical Traits of NAGLU Protein

Utilizing in silico methodologies, namely PredictSNP predictor, Meta-SNP, and SNPs&GO, a pathogenicity study was conducted to assess mutations affecting the protein. The obtained results classified the mutations as neutral, disease-related, or deleterious (Supplementary Table S2). Thirty-seven mutations were determined to be deleterious or disease-associated based on this research. From the above 37 pathogenic mutants, 29 mutants had a Class 65 property which denotes the most interfering mutants that affect the biophysical properties of the protein (Table 2, Supplementary Table S3).

### 2.3. Analyzing the Stability Change

The 29 mutations that are identified as the most pathogenic and were within the conserved regions, thereby affecting the protein’s biophysical function, were subjected to stability-based prediction. These mutants underwent stability change analysis using DUET (mCSM and SDM) and i-Stable (I-Mutant and MuPro) (Supplementary Table S4). Among these, nine mutations were observed to destabilize/decrease the stability, respectively (Table 2).

### 2.4. SNP Effects and HOPE Server Analysis

The SNPeffect analysis was conducted to analyze the phenotyping characterization of the above most pathogenic, highly conserved and most function-interfering destabilizing mutants (Supplementary Table S5). The results showed that the I154R and W649C mutants showed an increased tendency for chaperone binding and a severe reduction in protein stability and mutation, as shown in Table 3.

Based on the above findings, both the I154R and W649C mutants were analyzed using the HOPE tool (https://www.cmbi.umcn.nl/hope/, accessed on 15 March 2026). The I154R substitution resulted in the loss of hydrophobic interactions within the core region of the protein, as the mutant residue is larger than the wild type and does not fit optimally into the protein core. In contrast, the W649C variant replaces a bulky hydrophobic residue with a smaller one, likely creating an internal cavity and destabilizing the core structure. Both mutants are located in evolutionarily conserved regions and are predicted to be highly pathogenic. Therefore, further structure-based analyses focused on the I154R mutation were carried out (Table 4, Supplementary Table S6).

### 2.5. Investigating the Structure Retrieval

**Figure 1:**
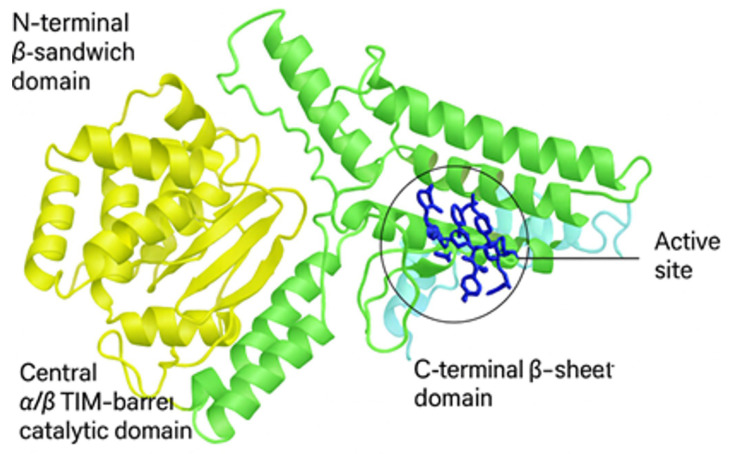
Structural representation of human α-N-acetylglucosaminidase (NAGLU, PDB ID: 4XWH). Ribbon diagram depicting the three-dimensional structure of human α-N-acetylglucosaminidase (NAGLU, PDB ID: 4XWH). The protein is composed of three distinct structural domains: the N-terminal β-sandwich domain (yellow), the central α/β TIM-barrel catalytic domain (green), and the C-terminal β-sheet domain (cyan). The active-site region is highlighted by a black circle and includes the key catalytic residues HIS408, ASN409, ASN413, HIS414, GLN450, ARG514, ASN526, GLU570, and ASP657, which definethe broader active-site region based on structural and functional annotation, displayed as blue sticks and labeled directly within the structure.

The crystal structure of human N-acetyl-alpha-glucosaminidase was obtained from the Protein Data Bank (PDB ID: 4XWH) with a resolution of 2.32 Å and an amino acid sequence range from 24 to 743. This structure showcases an asymmetric arrangement and monomer stoichiometry (Figure 1). The active-site cleft is located between domains II and III, with key catalytic residues, including glutamates E316 and E446, positioned within the (α/β) TIM-barrel. This structural organization highlights that substrate binding and catalysis occur within the monomeric enzyme, providing a basis for understanding mutation-induced functional alterations.

### 2.6. Molecular Interaction Analysis

The CASTp tool identifies the interacting sites within the protein structure with associated amino acid residues shown in Figure 1. Molecular docking was initiated in triplicate to analyze the molecular-level interaction between the protein and ligand using AutoDock software (v4.2, Table 5). Although N-acetylglucosamine (NAG) is present in the crystal structure (PDB ID: 4XWH), the ligand used for docking was retrieved from PubChem (CID: 439174) to ensure a standardized and geometry-optimized structure. The use of a curated ligand enables consistent preparation, energy minimization, and reproducibility in docking simulations and the scores were averaged for both native and mutant structures.

**Figure 2:**
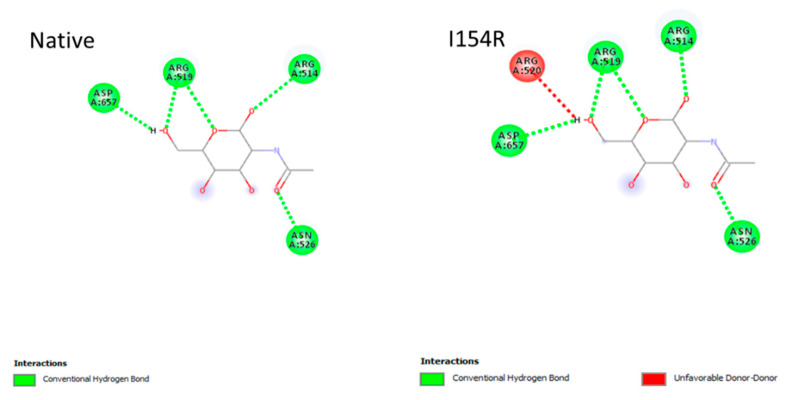
Molecular docking analysis of NAG binding to native and mutant (I154R) NAGLU. The figure illustrates the binding interactions of N-acetylglucosamine (NAG) within the active-site cleft of the native and mutant structures. Key interacting residues are highlighted, and hydrogen bonds are represented as dashed lines. Differences in binding orientation and interaction patterns between the native and mutant proteins are shown, indicating the impact of the I154R mutation on ligand binding.

Binding affinity values were obtained from multiple docking runs, and the results are presented as mean scores along with 95% confidence intervals (CI), calculated to assess the variability and reliability of the predicted interactions. The native structure exhibited a slightly stronger binding score of −4.17 ± 0.10 kcal/mol involving four conventional hydrogen bonds as the interacting amino acid residues (ARG514, ARG519, ASN526, and ASP657). In contrast, the mutant (I154R) displayed a weaker average score of −3.97 ± 0.30 kcal/mol, engaging five networking amino acid residues in which four (ARG514, ARG519, ASN526, and ASP657) amino acid residues are said to be conventional hydrogen bonds and one (ARG520) an unfavorable donor–donor which is not optimal for the ligand molecule (Figure 2). These active-site residues were defined based on structural proximity to the catalytic pocket, whereas the interacting residues were identified based on direct ligand contacts observed in docking.

### 2.7. Molecular Dynamics Simulation

**Figure 3:**
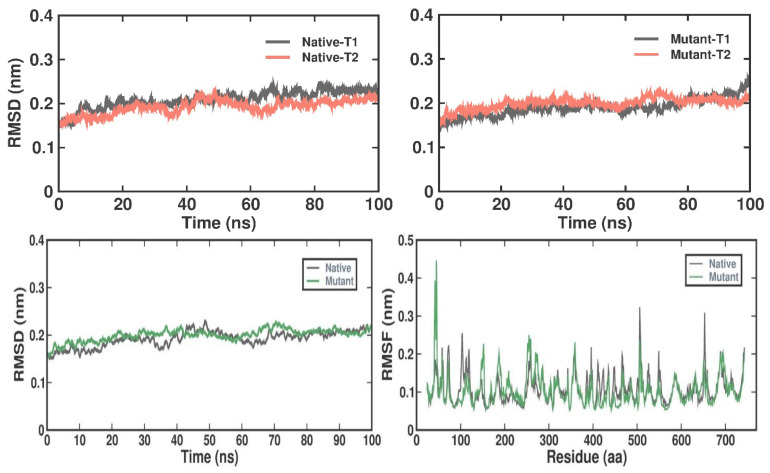
Block-based RMSD simulation runs with respect to both the native and mutant complexes for T1 and T2. T1 and T2 represent two independent molecular dynamics simulation trials (Trial 1 and Trial 2) performed for both native and mutant NAGLU systems to evaluate reproducibility and convergence of the simulation results. Conformational dynamics of RMSD and RMSF with respect to both the native and mutant complexes for T2 simulation run for a 100ns interval. Color representation: Gray—native and Green—I154R.

In this work, dynamic alterations that arise from target protein binding were examined using MD simulations. Trajectory parameters were computed for both the native and mutant–ligand complexes, including RMSD, RMSF, Rg, SASA, and hydrogen bonding along with principal component analysis and free-energy landscape analysis. The simulations were performed for two independent 100 ns simulation intervals. To assess the convergence of the simulation trials, block-based RMSD analysis was carried out between the two independent 100 ns simulations of the native and mutant complex structures. Notably, the second simulation run displayed a more favorable convergence range compared to the initial one (Figure 3).

We examined RMSD values over time to investigate the stability of the native and I154R complexes and clarify their dynamic behavior. Throughout the simulation period, both complexes demonstrated constant stability with little fluctuations. The native and mutant complexes had average RMSD values of 0.20 ± 0.01 nm and 0.22 ± 0.01 nm, both the structures converged at the end of the simulation interval. We used RMSF analysis to evaluate the variations in individual residues and flexible areas in the protein during MD simulations. This made it possible for us to look at how ligand binding affects the dynamics of proteins. According to our research, the RMSF distribution overall did not significantly change for the native or I154R complexes. The native and mutant complexes had average RMSF values of 0.12 ± 0.03 nm and 0.11 ± 0.04 nm, respectively (Figure 3).

**Figure 4:**
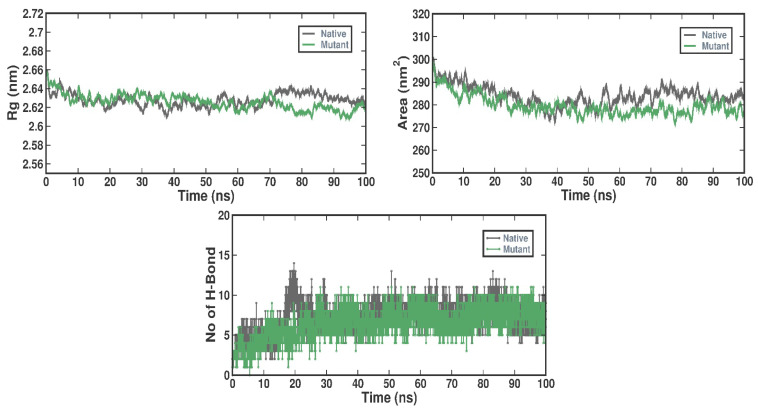
Conformational dynamics of Rg, SASA, and H-bond with respect to both the native andMutant complexes for T2 simulation run for 100ns interval. Color representation: Gray—native and Green—I154R.

To evaluate the dynamic stability and compactness of the native and I154R complexes, we computed and graphed the Rg values over a 100 ns simulation period. The average radius of gyration values for the native and mutant complexes were determined to be 2.62 ± 0.008 nm and 2.62 ± 0.009 nm, respectively. Assessing the accessibility of protein molecules in a solvent environment is essential, and SASA serves as a valuable parameter for this purpose. We calculated and plotted SASA values to discern the impact of I154R binding on native solvent accessibility. The average SASA values for the native and mutant complexes were 284.02 ± 5.22 nm^2^ and 279.61 ± 5.09 nm^2^, respectively. The formation of hydrogen bonds is crucial to gauge the stability of protein–ligand interactions. We analyzed and displayed the time-varying behavior of hydrogen bonding in both native and mutant proteins. According to analysis, the docked complexes were stabilized throughout the simulation from 3 to 12 hydrogen bonds with the native and 1 to 10 with the mutant (Figure 4).

### 2.8. Principal Component Analysis (PCA)

**Figure 5:**
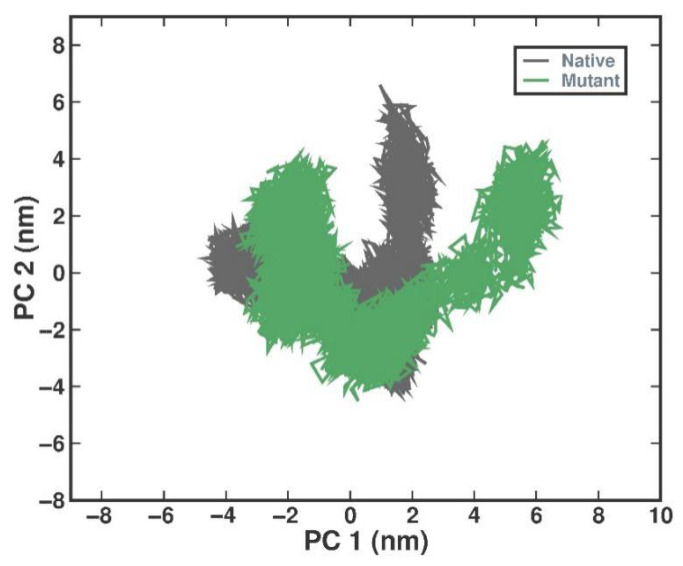
Principal component analysis 2D projection plot shows the conformation sampling of the native and mutant complexes on PC1 and PC2 in the T2 simulation run. Color representation: Gray—native and Green—I154R.

In order to examine the conformational dynamics of the native and I154R complexes during the simulation, PCA was utilized to look into group movements within them. The PCA’s temporal evolution analysis indicates a reduction in overall flexibility within the mutant complex on both eigenvectors (EVs), suggesting increased stability. The resulting plot illustrates a significant overlap between the conformational motions of the native and mutant complexes, indicating similar behavior (Figure 5).

### 2.9. MM-PBSA

The MM-PBSA binding energies are reported as mean ± standard deviation from frame-wise sampling over the final 50 ns of the trajectories. To assess the binding affinity of the native and I154R complexes, we investigated the relative binding strength within the protein by analyzing the summary energy. This analysis compares the binding strengths of the native and mutant complexes, along with the ligand, computed via the MM-PBSA method. Over a stable simulation trajectory, we calculated residue-level contributions to the interaction energy for the last 50 ns time interval (Table 6). The MM-PBSA analysis revealed a significant difference in binding free energy between the native (−59.24 ± 8.54 kJ/mol) and mutant (−11.66 ± 13.75 kJ/mol) complexes. This difference is primarily driven by an increase in polar solvation energy in the mutant system (276.91 ± 26.38 kJ/mol) compared to the native (241.80 ± 13.61 kJ/mol). Although the Van der Waals and electrostatic interactions remain relatively comparable, the elevated solvation penalty in the mutant reduces the overall binding affinity, indicating weaker ligand binding.

## 3. Discussion

Mutations in the NAGLU gene cause an excess of mucopolysaccharides (intralysosomal glycosaminoglycans) to accumulate in a variety of tissues [6,25]. The gene has been isolated and characterized, with numerous disease-causing mutations reported. While type B is phenotypically heterogeneous, with severe and mild forms compared to type A, most reported disease-causing mutations have been associated with the severe phenotype. Six different mutations were found to cause the condition, five of which were missense mutations and one of which was a two-base deletion [26]. In a prevalence rate analysis of mucopolysaccharidosis (MPSs) worldwide, the data from an average of 189 publications retrieved from PubMed as of December 2020 were collected and investigated. Compared to previous incidence rates, the frequency of MPS I rose after neonatal screening. It was determined that the identification techniques used now are inadequate to correctly identify every MPS patient, which results in erroneous estimations of incidence and status [27]. Regarding specific mutations, the study reported that among probands with Dutch ancestry, p.R344C and p.S518F were prevalent and accounted for 22.0% and 29.3% of the mutant alleles, respectively. Although other subtypes of MPS III, such as IIIC, display considerable clinical heterogeneity, our work specifically focused on NAGLU-related MPS IIIB, integrating structural and functional insights through computational pipelines [28].

The present study used a comprehensive computational pipeline to evaluate the potential impact of NAGLU mutants by integrating conservation profiling, pathogenicity scoring, and stability analysis. ConSurf analysis revealed that Ile154 lies within a highly evolutionarily conserved region of NAGLU, indicating strong functional constraint at this position. In contrast, several other pathogenic substitutions occur at comparatively less conserved sites, suggesting that they impair function primarily through global destabilization. The I154R mutation therefore represents a distinct mechanistic class, in which disruption of a highly conserved local microenvironment leads to loss of catalytic competence despite preservation of overall protein fold. From docking analysis, molecular dynamics (MD) simulations, and binding free-energy estimation (MM-PBSA), we identified consistent signals that I154R destabilizes the protein and reduces ligand-binding strength. This convergence across multiple independent tools highlights the predictive value of in silico analysis for understanding disease-associated mutants in MPS IIIB. The crystal structure of human N-acetyl-α-D-glucosaminidase (PDB ID: 4XWH) demonstrates that the catalytic machinery is contained within a single monomeric unit. This structural organization highlights that substrate binding and catalysis occur within the monomeric enzyme, providing a basis for understanding mutation-induced functional alterations [13] RCSB PDB 4XWH. Therefore, modeling the monomer is appropriate for probing substrate binding and dynamics at the active site, consistent with common practice in early-stage docking and molecular dynamics pipelines [29,30].

Although the I154R mutant retained a docked N-acetylglucosamine binding pose within the active site, this does not imply preserved enzymatic function. Docking primarily reports on ligand accommodation within the binding pocket, whereas protein stability and catalytic competence depend on the broader structural integrity and precise local geometry of active-site residues. Subsequent molecular dynamics analyses therefore evaluated both global structural stability and local active-site rearrangements to distinguish between maintained ligand binding and functional competence. The introduction of a positively charged residue at a buried hydrophobic position (Ile→Arg) perturbs packing, reduces thermodynamic stability, and increases the likelihood of misfolding or premature degradation. In a cellular context, such instability is expected to lower the steady-state concentration of a correctly folded enzyme reaching the lysosome, thereby reducing overall catalytic throughput even if some binding potential is transiently retained. This mechanistic model is consistent with clinical observations in MPS IIIB, where missense mutations often lead to residual activity that is insufficient to prevent heparan sulfate accumulation and progressive neurodegeneration [31].

NAGLU functions within the acidic environment of the lysosome. However, the present simulations were conducted using standard protonation states corresponding to neutral pH. While acidic pH can modulate electrostatic interactions, the relative comparison between wild-type and I154R structures remains valid because both were evaluated under identical conditions. Importantly, mutation-induced perturbations in substrate positioning and active-site geometry are driven primarily by local structural rearrangements, which are preserved across pH states. Future constant-pH or pH-explicit molecular dynamics simulations will be valuable to further refine these findings under fully lysosomal conditions [32].

The MD simulations revealed only modest deviations in RMSD, RMSF, and compactness; however, these subtle shifts were consistent across independent analyses, including docking and MM/PBSA free-energy calculations. In lysosomal enzymes, where catalytic activity depends on precise folding and microenvironmental stability, even small perturbations can significantly impair function [16,17,18]. Molecular dynamics simulations combined with MM-PBSA analysis have been widely employed to investigate protein–ligand interactions and mutation-induced structural effects, providing reliable insights into binding stability and conformational dynamics [33,34,35,36]. Therefore, the reproducibility and convergence of our computational findings suggest that the I154R mutation exerts a biologically relevant destabilizing effect, despite appearing modest at the structural scale. This insight suggests the capacity of computational pipelines to prioritize mutants of possible structural and functional importance. Our analysis of the I154R variant demonstrates that even rare mutations can inform future genotype–phenotype studies in Sanfilippo syndrome type B.

### Limitations and Experimental Outlook

The present study is computational in nature and is designed to provide atomistic-level mechanistic insight into how the I154R mutation perturbs NAGLU function. While direct enzymatic assays such as over-expression of wild-type and mutant NAGLU followed by activity measurement would provide valuable functional confirmation, such experiments require specialized lysosomal targeting systems and patient-derived cellular models that are beyond the scope of this work. Importantly, molecular modeling enables the identification of subtle but functionally critical disruptions in substrate binding and catalytic geometry that may not be apparent from global activity measurements alone. Future experimental studies combining enzyme kinetics, lysosomal localization assays, and patient-derived fibroblasts will be valuable to further validate the mechanistic predictions reported here.

## 4. Materials and Methods

### 4.1. Mutation Dataset and Structure Recovery from Public Domains

The *NAGLU* gene mutations were recovered from databases like NCBI (National Center for Biotechnology Information (NCBI), Bethesda, MD, USA (https://www.ncbi.nlm.nih.gov/)) (ClinVar), HGMD (Human Gene Mutation Database (HGMD), Cardiff University, Cardiff, UK (http://www.hgmd.cf.ac.uk/)) and UniProt (UniProt Consortium, European Bioinformatics Institute (EMBL-EBI), Hinxton, Cambridge, UK (https://www.uniprot.org/)). The UniProt database was used to obtain the protein sequence (ID- P54802) with a length of 743 amino acids, and the FASTA input was applied for further analysis. The crystal structure of human NAGLU, with a resolution of 2.3 Å, lacks the N-terminal carbohydrate-binding module (CBD) and has a 28.2% sequence identity with the bacterial homolog, CpGH89, across a 692-amino-acid overlap. Like the bacterial enzyme, NAGLU has three different domains in its structure. Domain I (residues 24–126), a short α/β domain at the bottom of the catalytic pocket that is sandwiched between helical domain III (residues 466–743) and catalytic domain II (residues 127–465) by a two-stranded β-sheet [13]. The missing residues were worked upon, and the structure was optimized and energy-minimized with the help of the Swiss PDB viewer 4.1.0. The wild-type structure was mutated using PyMOL version 2.4 software [37].

### 4.2. Identifying the Conserved Region of the NAGLU Protein

The ConSurf tool was used to give insight into the vital domain that hinders the functional activity of the protein. The multiple sequence alignment was performed against the UNIREF90 database. A Bayesian substitution model was applied. ConSurf assigns conservation scores ranging from 1 (highly variable) to 9 (highly conserved). Residues with scores of 1–4 are considered variable to moderately variable, scores of 5 indicate an intermediate (average) level of conservation, and scores of 6–9 represent increasingly conserved positions and those with a score of 9 were considered to lie in highly conserved regions [38].

### 4.3. Investigating the Pathogenicity of the Mutant Proteins

The mutagenic analysis was converged to analyze and classify the mutations that are likely to be a cause of threat to a specific disease. Thepathogenicity method comprises the following tools: 1: SNPs&GO shows the mutagenic outcome of the protein mutants. It works with the help of SVM, which categorizes and investigates the mutations, and it creates five different tool outputs, i.e., PhD-SNP, SIFT, PANTHER, SNAP, and SNPs&GO. 2: Meta-SNP combines four methods: PANTHER, PhD-SNP, SIFT, and SNAP [39]. 3: Predict SNP predictor is a web interface that allows easy access to all eight prediction tools (MAPP, nsSNPAnalyzer, PANTHER, PhD-SNP, PolyPhen-1, PolyPhen-2, SIFT, SNAP). The six best-performing tools were combined into a consensus classifier PredictSNP that gives an outcome resulting from the considerably enhanced prediction and gives accurate and robust predictions delivered by individual tools [40]. These pathogenic techniques forecast the variation’s neutral or damaging characteristics.

### 4.4. Characterizing the Biophysical Nature

The align-GVGD allows calculation of the mutations with extremely distant amino acids and their origin of effects. It is predominantly performed to understand whether it is vastly damaging or does not cause damage to the protein function [41]. The output gives a varied range of classes from 0 to 65, which denotes the likelihood that it may affect the protein function.

### 4.5. Identifying the Stable Nature of the Mutant Proteins

Protein mutations can cause physical changes and can even cause disease. The results of a stability analysis indicate whether a protein structure is more likely to be destabilizing or stabilizing. The in silico procedures listed below are used to measure the change in the stability: 1: iStable tool that combines with two other tools, namely, I mutant and MUpro [42]. The integrated tool predicts variations in protein stability resulting from mutations in a particular amino acid residue using a support vector machine (SVM). 2: DynaMut employs integrated tools, namely DUET, mCSM, and SDM, to understand the structural stability of the protein structure for enhanced prediction. The methodology was based on measuring the vibrational entropy [43].

### 4.6. Phenotyping Analysis and HOPE Server for the NAGLU Protein

The SNPeffect aids in investigating the impact of SNP on the phenotypic characteristics of human proteins [44]. Four outputs have been added to the SNPeffect 4.0 server: WALTZ predicts the amyloid propensity, FOLDX determines the protein stability, LIMBO determines the chaperone binding propensity, and TANGO understands the aggregation prediction [45]. HOPE is a comprehensive automated program designed to analyze both the structural and functional implications of point mutations. It gathers data from diverse sources, including calculations based on the protein’s 3D coordinates utilizing predictions from DAS services, sequence annotations from the UniProt database, WHAT IF Web services, and YASARA to create homology models [46].

### 4.7. Protein–Ligand Interaction Analysis

To determine the binding profile of the native and mutant protein structures, molecular docking was performed using AutoDock v4.2 [47,48,49]. The active-site region of the protein was identified using the Computed Atlas of Surface Topography of Proteins (CASTp 3.0), which detects surface pockets, internal cavities, and functional binding sites [50]. The crystal structure of N-acetyl-α-D-glucosaminidase (PDB ID: 4XWH) and the mutant (I154R) model were prepared by removing water molecules, adding polar hydrogen atoms, and assigning Kollman and Gasteiger charges. The ligand, N-acetylglucosamine (NAG), was energy-minimized and converted into pdbqt format. A grid box was defined around the active-site region with dimensions of 67 × 73 × 64 points along the x, y, and z axes, respectively, with a grid spacing of 0.370 Å. Docking simulations were performed using the Lamarckian Genetic Algorithm (LGA) with 100 runs per ligand, a population size of 150, and a maximum of 2,500,000 energy evaluations. Default parameters were used for the remaining settings [51,52]. Docking was performed in three independent trials, and the average binding affinity values were calculated for both the native and mutant complexes. The resulting docked conformations were analyzed based on binding energy and root mean square deviation (RMSD). The protein–ligand interactions and binding modes were visualized using Discovery Studio Visualizer version 4.5 [53].

### 4.8. Molecular Dynamics Simulation

The native and mutant protein–ligand complexes were simulated using the GROMACS version 2020.2 version molecular dynamics package, employing the GROMOS 54a7 force field to describe the interatomic interactions. The docked configuration exhibiting the most favorable binding energy (lowest value) was selected as the initial structure for the simulations. The PRODRG web server was used to construct the ligand topology; NAG_PRODRG.itp provides the atom table, empirical partial atomic charges, and bonded parameter sections ([bonds], [angles], and [dihedrals]).These PRODRG-derived charges and bonded parameters were used without further modification and mapped directly to the GROMOS 54a7 protein force field used in this study and they are provided in the Supplementary Materials [54,55]. The complexes were solvated in a dodecahedron box of simple point charge (SPC) water molecules, ensuring a sufficient solvation shell around the solute. Counter ions were introduced by substituting water molecules to neutralize the overall charge of the simulated system. Energy minimization was performed to alleviate steric clashes and optimize the structures. Subsequently, a two-step equilibration protocol was employed. Initially, a 300 picosecond (ps) NVT (constant number of particles, volume, and temperature) equilibration was conducted, gradually increasing the temperature to 300 K to achieve thermal equilibration. This was followed by a 300 ps NPT (constant number of particles, pressure, and temperature) equilibration to stabilize the system’s pressure and density. The equilibrated configurations were then subjected to production molecular dynamics simulations for two independent 100 ns simulations (ns), as recommended by [18] for robust sampling of the conformational ensemble. This simulation protocol, incorporating state-of-the-art force fields, solvation models, and equilibration procedures, is designed to capture the intricate dynamics and interactions of protein–ligand combinations, understanding the stability, conformational transitions, and binding mechanisms at an atomistic level.

### 4.9. Trajectory Analysis, Principal Component Analysis and Binding Free-Energy Calculations (MM-PBSA)

A variety of GROMACS utilities were used to do the trajectory analysis: gmx rmsd for root mean square deviation, gmx rmsf for root mean square fluctuation, gmx gyrate for radius of gyration, gmx sasa for solvent-accessible surface area, and gmx hbond for hydrogen-bond work. The trajectories were obtained in the .xtc format. Principal component analysis (PCA) was conducted to investigate the slow and functionally relevant motions of the complex components. The gmx covar utility was employed to perform PCA, and gmx anaeig was implemented to extract the eigenvectors and eigen values [56]. As an output, the RMS fluctuation per atom of the eigenvectors was computed. A graphic representation was [57] used to show the protein trajectory’s first and second principal component projections. The MM-PBSA methodology uses molecular mechanics to assess, during the simulation period, the binding free energy (ΔG binding) between the ligand molecules and the target proteins. The binding free energy was evaluated using the g_mmpbsa GROMACS plugin. For the final 50 nanoseconds (ns) of the simulation, MM-PBSA analysis was carried out [58,59]. In order to achieve a thorough comprehension of both native and mutant protein structures, the simulation data from the duplicate 100 ns trajectories were plotted using Qtgrace version 0.2.6 [60]. This comprehensive trajectory analysis protocol, incorporating various GROMACS utilities and advanced techniques such as PCA and MM-PBSA, enables the investigation of structural dynamics, conformational changes, functional motions, and binding interactions of the protein–ligand complexes at an atomistic level, providing valuable insights into their molecular and energetic behavior.

## 5. Conclusions

Through a comprehensive computational pipeline, this study aimed to identify significant NAGLU mutations using publicly available datasets. The focus was on the I154R mutation, predominantly situated in conserved regions and deemed highly pathogenic. This mutation was observed to destabilize the protein structure and interfere with its functionality while also exhibiting important phenotyping properties. The subsequent step involved checking its interaction profiling strategy and interatomic simulation, which resulted in notable interaction scores of −4.19 kcal/mol for the native protein and −3.97 kcal/mol for the ligand (NAG). Trajectory analyses confirmed these findings, indicating stable overall dynamics, consistent flexibility, compactness, and favorable hydrogen bond formation within the protein–ligand complex. MMPBSA scores also corroborated the docking results. This study provides computational insights that may support the development of future treatment strategies for Sanfilippo syndrome type B (MPS IIIB), particularly concerning NAGLU proteins.

## Figures and Tables

**Table 1:** Datasets retrieved from publicly available databases (ClinVar, UniProt, and HGMD).

Protein Name	Dataset Information			
	ClinVar	UniProt	HGMD	Total nsSNP’s
Alpha-N-acetylglucosaminidase (NAGLU)	54	68	140	162

**Table 2:** Number of amino acid mutants scrutinized from each analysis (ConSurf, pathogenicity, Align GVGD, stability analysis) for NAGLU protein.

Mutational Analysis	No. of Amino Acid Mutants
Conservational analysis (ConSurf)	45 mutants
Pathogenicity prediction (PredictSNP predictor, SNPs&GO, Meta-SNP, Panther, PHD-SNP, SIFT, SNAP)	37 mutants
Biophysical characteristics (Align GVGD)	29 mutants
Structural stability (Dynamut, Mcsm, SDM, DUET, and i-Stable (i-Mutant and MuPro)	9 mutants

**Table 3:** Effects of highly deleterious SNPs on NAGLU protein aggregation, amyloid propensity, chaperone binding, and protein stability.

Database ID(s)	Amino Acid Variant	TANGO (Aggregation)	WALTZ (Amyloid)	LIMBO (Chaperone Binding)	FOLDX (Stability)
VAR_054707	I154R	The mutation affects the aggregation tendency of the protein.	The mutation does not affect the amyloid propensity of the protein.	The mutation increases the chaperone binding tendency of the protein.	The mutation severely reduces protein stability.
VAR_054735, CM003011	W649C	The mutation does not affect the aggregation tendency of the protein.	The mutation does not affect the amyloid propensity of the protein.	The mutation increases the chaperone binding tendency of the protein.	The mutation severely reduces protein stability.

**Table 4:** Predicted structural and functional effects of NAGLU mutations based on HOPE server analysis.

RSID	Mutation	Hydrophobicity/Core Effect	Size Change	Location	Pathogenicity Score	HOPE Significance
VAR_054707	I154R	Loss of hydrophobic interactions (core)	Mutant larger than wild type	Wild type buried in core	0.9907	Highly significant: Core residue, hydrophobic loss, size clash, high conservation
VAR_054735, CM003011	W649C	Not reported	Mutant smaller than wild type	Core (creates empty space)	0.9964	Significant: Core residue, space created, high conservation

**Table 5:** Overall triplicate docking scores for native and I154R mutant NAGLU protein structures complexes with the NAG ligand. Values are presented as individual docking runs (D1–D3), with average docking score, standard deviation (SD), and 95% confidence interval (CI) in kcal/mol.

Mutations	D1	D2	D3	Average Docking Score	SD	95% CI (kcal/mol)
Native	−4.24	−4.21	−4.06	−4.17	−4.17 ± 0.10	−4.41 to −3.93
I154R	−4.29	−3.93	−3.69	−3.97	−3.97 ± 0.30	−4.72 to −3.22

**Table 6:** The binding energy score achieved from the MMPBSA package of the GROMACS for the T2 simulation run.

Protein Complexes	Van Der Waals Energy	Electrostatic Energy	Polar Solvation Energy	Binding Energy
Native	−27.612 ± 9.432 kJ/mol	−262.302 ± 11.432 kJ/mol	241.798 ± 13.609 kJ/mol	−59.237 ± 8.539 kJ/mol
I154R	−25.752 ± 22.626 kJ/mol	−253.380 ± 28.895 kJ/mol	276.907 ± 26.381 kJ/mol	−11.664 ± 13.747 kJ/mol

## Data Availability

The original contributions presented in this study are included in the article/Supplementary Materials. Further inquiries can be directed to the corresponding authors.

## Supplementary Materials

The following supporting information can be downloaded at https://www.mdpi.com/article/10.3390/ijms27104404/s1.

## Abbreviations

The following abbreviations are used in this manuscript:

LSD	Lysosomal storage diseases
MPS IIIB	Mucopolysaccharidosis IIIB
NAGLU	α-N-acetylglucosaminidase
GVGD	Grantham variant and Grantham difference
PROVEAN	Protein variation effect analyzer
PANTHER	Protein analysis through evolutionary relationships
SIFT	Sorting intolerant from tolerant
PCA	Principle component analysis
MM/PBSA	Molecular mechanics Poisson–Boltzmann surface area
RMSD	Root mean square deviation
RMSF	Root mean square fluctuation
H-Bond	Hydrogenbond
