# Supplementary material for: Molecular Modeling of N-Acetylglucosamine Binding to the I154R Mutant of NAGLU: Pathogenic Insights into Sanfilippo Syndrome Type B
Source: Int J Mol Sci. 2026 May 15;27(10):4404. doi: 10.3390/ijms27104404 (PMC13207710; doi:10.3390/ijms27104404)
Supplement: Supplementary file 1 [file ijms-27-04404-s001.zip › ijms-4231592-supplementary.pdf]

Molecular Modeling of N-Acetylglucosamine Binding to the I154R Mutant of NAGLU: Pathogenic Insights into Sanfilippo Syndrome Type B

Priyanka Kannan[1], Madhana Priya Nanda Kumar[1], Sidharth Kumar Nanda Kumar[1], Vasundra Vasudevan[1], Kuppan Kaviarasan [2,*] and Magesh Ramasamy [1,*]

[1] Department of Biotechnology, Sri Ramachandra Institute of Higher Education and Research (DU), Porur 600116, Chennai, TamilNadu, India.
[2] Department of Biomedical Sciences, Sri Ramachandra Institute of Higher Education and Research (DU), Porur 600116, Chennai, TamilNadu, India.
* Correspondence: kkavi@sriramachandra.edu.in; magesh.r@sriramachandra.edu.in

Supplementary Table S1: overall results of both dataset and conservational analysis

Rsid, HGMD, UniProt	Mutations	ConSurf
1458683	M1I	-
CM080445	M1K	-
CM990914	M1L	-
553224	M1T	-
488848	M1V	-
CM1815245	A9E	
VAR_054699,1490129,CM003004	L35F	6
CM1617764	L35P	6
VAR_054700,CM053337	R38W	9
VAR_054701,CM981354	F48C	8
VAR_025489,1569,CM992348	F48L	8
CM108284	D63N	7
CM134534	L67P	5
VAR_054702,CM992349	G69S	6
CM128257	G70R	1
VAR_054703,638092,CM053340	V77G	7
VAR_008979,CM990915	G79C	8
VAR_054704	G79S	8
VAR_054705,803391,CM003005	G82D	5
BM1233579	A87P	6
VAR_005007,CM981355	Y92H	9
973593	C97W	5
VAR_008980,CM990916	H100R	6
VAR_005008,553505,CM981356	P115S	9
1710486	N127K	6
VAR_054706,CM023433	R130C	8

CM1823489	R130H	8
CM1823478	Y131S	9
VAR_005009,371634,CM981357	Y140C	9
1481925	Y140N	9
CM128258	S141F	8
VAR_005010,1067431,CM981358	E153K	8
VAR_054707	I154R	9
CM1823467	I154T	9
VAR_054708,CM003006	W156C	9
CM128259	G161D	9
CM166990	G170D	9
CM1823507	G183A	8
CM108098	R177W	5
973948,CM187395	P196L	9
CM1922698	T209P	5
VAR_054709,280994,CM992350	H227P	5
VAR_054710,1572,CM981360	R234C	7
1013390	R234G	7
CM1823488	G237D	7
VAR_054711,CM023646	V241M	8
VAR_054712,CM040463	L242P	8
VAR_008982,CM990917	P243L	8
VAR_054713,CM053339	A246P	7
1710495	G247W	9
VAR_054714,CM992351	H248R	8
VAR_054715,CM981361	W268R	9
CM1823502	S276F	6
VAR_008983,CM990918	C277F	5
CM1823482	S278F	6
VAR_008984,CM990919	L280P	6
CM1823496	L281P	8
CM108285	A282V	6
VAR_008985,CM990920	G292R	9
1511855	G292E	9
803392,CM080447	G304V	9
VAR_054716,841655,CM023435	Y309C	9
1473772	Y309H	9
841655	Y309F	9
546392	G310R	8
CM1823491	G310V	8
CM113463	D312N	9

VAR_025490,1570,CM023647,CM166234	F314L	8
VAR_054717,1345828	F314S	8
VAR_054718,1495526	F314V	8
CM1823508	L327F	8
CM992352	V334F	7
803393	Y335S	5
CM053341	Y335C	5
CM1617766	M338V	8
CM1823486	T339P	4
CM1823499	L349F	6
CM1823494	F354S	9
VAR_005011,CM981362	P358L	3
1067432,CM130686	W361R	9
CM1823503	W361S	9
CM1823479	R377H	6
CM192272	D382Y	9
CM1823472	A385V	6
CM1823466	E386K	8
CM108286	Y391C	8
CM1823468	G399V	8
204585,CM108287	I403T	7
CM1823516	W404C	7
CM184200	H408Q	7
VAR_054719,CM992353	F410S	8
VAR_054720,CM023436	G412E	9
VAR_054721,552833,CM992354	H414R	2
1474299,CM1823500	G426A	3
643204	G426D	3
CM1823465	A430T	8
CM1823490	R431C	6
VAR_054722,CM020161	T437I	7
623313	M438R	8
CM128260	T441K	7
CM1823474	A444D	8
CM1823484	P445S	7
VAR_054723,CM040464	E446K	9
VAR_008986,871076,CM990922	E452K	8
CM1823477	V454F	6
VAR_054724,CM981363	Y455C	8
CM1823483	Y455S	8
CM1823476	G462R	6

CM1823470	R464Q	6
VAR_054725,CM010940	W474G	9
CM1823501	R481W	6
VAR_054726,CM040465	R482Q	9
VAR_008987,1571	R482W	9
CM1823492	L496P	4
830367,CM108288	L497V	9
CM1815242	L498V	4
VAR_054727,CM003008	V501G	7
CM1823487	N513S	5
VAR_054728,CM040466	P516L	6
VAR_054729,553515,CM003009	R520W	7
VAR_025491,1566,CM990923,CM981364	P521L	9
CM080448	S522P	6
VAR_054730,CM003010	S534Y	1
CM1617768	R541W	2
CM1823471	L542P	4
VAR_079425,CM173555	L550P	2
1324776,CM1823480	D559N	9
CM1823493	D559H	6
VAR_054731,CM992356	L560P	8
VAR_008988,1503288,CM990924	L561R	9
CM1823498	L563H	9
CM134818	R565L	9
VAR_025492,1568,CM992357	R565P	9
VAR_008989,30795,CM990925	R565Q	8
VAR_025493,1567,CM981365	R565W	6
CM175634	Q569K	7
CM113466	A582P	6
VAR_054732,CM981366	L591P	5
554402,CM118226	P604L	1
1489488	P604S	1
CM128261	A611P	5
VAR_054733,522823,CM981367	S612G	7
CM184199	R615C	5
VAR_054734,CM992358	L617F	6
CM1617769	L622P	6
CM1823497	E634K	7
VAR_025494,1565,CM992359	R643C	8
VAR_005012,CM961017	R643H	8
CM1823495	Q645P	8

VAR_054735,CM003011	W649C	9
CM1823475	W649S	9
143187,CM137951	W649L	9
VAR_054736,143188,CM992360	G650E	9
VAR_054737,CM010941	Y658F	9
572445	Y658C	9
VAR_005013,521840,CM981368	A664V	3
VAR_054738,553061,CM981369	R674C	9
VAR_005014,1560,CM950832	R674H	9
VAR_054739,CM992361	R676P	3
VAR_005015,CM981371	L682R	5
VAR_008990,CM981372	E705K	9
CM1823504	K729R	1

Supplementary Table S2: overall results of pathogenicity prediction

rsid, HGMD, UniProt	Mutations	PANTHER	PhD-SNP	SIFT	SNAP	Meta-SNP	SNP&GO	predict SNP	MAPP
VAR_054700,CM053337	R38W	**D**	**D**	**D**	**D**	**D**	**D**	**D**	**D**
VAR_005007,CM981355	Y92H	**D**	**D**	**D**	**D**	**D**	**D**	**D**	**D**
VAR_005008,553505,CM981356	P115S	**D**	**N**	**D**	**D**	**N**	**D**	**D**	**D**
VAR_005009,371634,CM981357	Y140C	**D**	**D**	**D**	**D**	**D**	**D**	**D**	**D**
1481925	Y140N	**D**	**D**	**D**	**D**	**D**	**D**	**D**	**D**
VAR_054707	I154R	**D**	**D**	**D**	**D**	**D**	**D**	**D**	**D**
CM1823467	I154T	**D**	**D**	**D**	**D**	**D**	**D**	**D**	**D**
VAR_054708,CM003006	W156C	**D**	**D**	**D**	**D**	**D**	**D**	**D**	**D**
CM128259	G161D	**D**	**D**	**D**	**D**	**D**	**D**	**D**	**D**
CM166990	G170D	**D**	**D**	**D**	**D**	**D**	**D**	**D**	**D**
973948,CM187395	P196L	**D**	**D**	**D**	**D**	**D**	**D**	**D**	**D**
1710495	G247W	**D**	**D**	**D**	**D**	**D**	**D**	**D**	**D**

VAR_054715,CM981361	W268R	**D**	**D**	**D**	**D**	**D**	**D**	**D**	**D**
VAR_008985,CM990920	G292R	**N**	**D**	**D**	**D**	**D**	**D**	**D**	**D**
1511855	G292E	**D**	**D**	**D**	**D**	**D**	**D**	**D**	**D**
803392,CM080447	G304V	**D**	**D**	**D**	**D**	**D**	**D**	**D**	**D**
VAR_054716,841655,CM023435	Y309C	**D**	**D**	**D**	**D**	**D**	**D**	**D**	**D**
1473772	Y309H	**D**	**D**	**D**	**D**	**D**	**D**	**N**	**N**
841655	Y309F	**N**	**D**	**N**	**D**	**D**	**D**	**D**	**D**
CM113463	D312N	**D**	**D**	**D**	**D**	**D**	**D**	**D**	**D**
CM1823494	F354S	**D**	**D**	**D**	**D**	**D**	**D**	**D**	**D**
1067432,CM130686	W361R	**D**	**D**	**D**	**D**	**D**	**D**	**D**	**D**
CM1823503	W361S	**D**	**D**	**N**	**D**	**D**	**D**	**D**	**D**
CM192272	D382Y	**D**	**D**	**N**	**D**	**D**	**D**	**D**	**D**
VAR_054720,CM023436	G412E	**D**	**D**	**D**	**D**	**D**	**D**	**D**	**D**
VAR_054723,CM040464	E446K	**D**	**D**	**D**	**D**	**D**	**D**	**D**	**D**
VAR_054725,CM010940	W474G	**D**	**D**	**D**	**D**	**D**	**D**	**D**	**D**
VAR_054726,CM040465	R482Q	**D**	**D**	**D**	**D**	**D**	**D**	**D**	**D**
VAR_008987,1571	R482W	**D**	**D**	**D**	**D**	**D**	**D**	**D**	**D**
830367,CM108288	L497V	**D**	**D**	**D**	**D**	**D**	**D**	**D**	**D**
VAR_025491,1566,CM990923,CM981364	P521L	**D**	**D**	**N**	**D**	**D**	**D**	**D**	**D**
1324776,CM1823480	D559N	**D**	**D**	**D**	**D**	**D**	**D**	**D**	**D**
VAR_008988,1503288,CM990924	L561R	**D**	**D**	**D**	**D**	**D**	**D**	**D**	**D**
CM1823498	L563H	**D**	**D**	**N**	**N**	**D**	**D**	**D**	**D**
CM134818	R565L	**D**	**D**	**N**	**D**	**D**	**D**	**D**	**D**

VAR_025492,1568,CM992357	R565P	**D**	**D**	**D**	**D**	**D**	**D**	**D**	**N**
VAR_054735,CM003011	W649C	**D**	**D**	**D**	**D**	**D**	**D**	**D**	**D**
CM1823475	W649S	**D**	**D**	**D**	**D**	**D**	**D**	**D**	**D**
143187,CM137951	W649L	**D**	**D**	**D**	**D**	**D**	**D**	**D**	**D**
VAR_054736,143188,CM992360	G650E	**D**	**D**	**D**	**D**	**D**	**D**	**D**	**D**
VAR_054737,CM010941	Y658F	**D**	**D**	**D**	**D**	**D**	**D**	**D**	**D**
572445	Y658C	**D**	**D**	**D**	**D**	**D**	**D**	**D**	**D**
VAR_054738,553061,CM981369	R674C	**D**	**D**	**D**	**D**	**D**	**D**	**D**	**D**
VAR_005014,1560,CM950832	R674H	**D**	**D**	**D**	**D**	**D**	**D**	**D**	**D**
VAR_008990,CM981372	E705K	**D**	**D**	N	**D**	**D**	**D**	**D**	N

[D- disease/ deleterious, N- neutral]

Supplementary table S3 overall results of Align-GVGD analysis

**Rsid, HGMD, UniProt**	**Mutations**	**Align GVGD**
VAR_054700,CM053337	R38W	Class C65
VAR_005007,CM981355	Y92H	Class C65
VAR_005009,371634,CM981357	Y140C	Class C65
1481925	Y140N	Class C65
VAR_054707	I154R	Class C65
CM1823467	I154T	Class C65
VAR_054708,CM003006	W156C	Class C65
CM128259	G161D	Class C65
CM166990	G170D	Class C65
973948,CM187395	P196L	Class C65
1710495	G247W	Class C65
VAR_054715,CM981361	W268R	Class C65
1511855	G292E	Class C65
803392,CM080447	G304V	Class C65

VAR_054716,841655,CM023435	Y309C	Class C65
CM113463	D312N	Class C15
CM1823494	F354S	Class C65
1067432,CM130686	W361R	Class C65
CM1823503	W361S	Class C65
CM192272	D382Y	Class C65
VAR_054720,CM023436	G412E	Class C65
VAR_054723,CM040464	E446K	Class C55
VAR_054725,CM010940	W474G	Class C65
VAR_054726,CM040465	R482Q	Class C35
VAR_008987,1571	R482W	Class C65
830367,CM108288	L497V	Class C25
1324776,CM1823480	D559N	Class C15
VAR_008988,1503288,CM990924	L561R	Class C65
CM1823498	L563H	Class C65
VAR_054735,CM003011	W649C	Class C65
CM1823475	W649S	Class C65
143187,CM137951	W649L	Class C55
VAR_054736,143188,CM992360	G650E	Class C65
VAR_054737,CM010941	Y658F	Class C15
572445	Y658C	Class C65
VAR_054738,553061,CM981369	R674C	Class C65
VAR_005014,1560,CM950832	R674H	Class C25

Supplementary table S4 overall results of stability prediction

rsid, HGMD, UniProt	Mutations	Dynamut	sdm	duet	MCSM	**i-Mutant**	**MUpro**	**iStable**
VAR_054700, CM053337	R38W	D	S	D	D	D	D	D
VAR_005007, CM981355	Y92H	D	D	D	D	D	D	D
VAR_005009, 371634,CM981357	Y140C	D	D	D	D	D	I	I

1481925	Y140N	D	D	D	D	D	I	I
VAR_054707	I154R	D	D	D	D	D	D	D
CM1823467	I154T	D	D	D	D	D	D	D
VAR_054708, CM003006	W156C	D	D	D	D	D	D	D
CM128259	G161D	D	D	D	D	D	D	D
CM166990	G170D	D	D	D	D	D	I	I
973948,CM187395	P196L	S	S	S	D	I	D	I
1710495	G247W	D	D	D	D	I	D	D
VAR_054715, CM981361	W268R	D	D	D	D	D	I	I
1511855	G292E	S	D	D	D	D	I	I
803392,CM080447	G304V	S	D	D	S	D	D	D
VAR_054716, 841655,CM023435	Y309C	D	D	D	D	I	D	D
CM1823494	F354S	D	D	D	D	D	D	D
1067432,CM130686	W361R	D	D	D	D	D	D	D
CM1823503	W361S	D	D	D	D	D	D	D
CM192272	D382Y	S	S	D	D	D	D	D
VAR_054720, CM023436	G412E	S	D	D	D	D	D	D
VAR_054725, CM010940	W474G	D	D	D	D	D	NULL	D
VAR_008987, 1571	R482W	S	S	D	D	I	D	I
VAR_008988, 1503288,CM990924	L561R	D	D	D	D	I	NULL	I
CM1823498	L563H	D	D	D	D	D	NULL	D
VAR_054735, CM003011	W649C	D	D	D	D	D	D	D
CM1823475	W649S	S	D	D	D	NULL	NULL	D
VAR_054736, 143188,CM992360	G650E	D	D	D	D	D	I	I
572445	Y658C	D	D	D	D	D	NULL	D

VAR_054738, 553061,CM98 1369	R674C	D	D	D	D	D	NULL	D

[D- Destabilizing/decrease, S- Stabilizing, I- Increase]

Supplementary table S5 overall results of Phenotyping analysis

rsid, HGMD, UniProt	Mutations	TANGO	WALTZ	LIMBO	FOLDX
VAR_005007,CM981355	Y92H	The mutation does not affect the aggregation tendency of your protein.	The mutation does not affect the amyloid propensity of your protein.	The mutation does not affect the chaperone binding tendency of your protein.	The mutation reduces the protein stability.
VAR_054707	I154R	The mutation does not affect the aggregation tendency of your protein	The mutation does not affect the amyloid propensity of your protein.	The mutation increases the chaperone binding tendency of your protein.	The mutation severely reduces the protein stability.
CM1823467	I154T	The mutation does not affect the aggregation tendency of your protein.	The mutation does not affect the amyloid propensity of your protein.	The mutation increases the chaperone binding tendency of your protein	The mutation reduces the protein stability.
VAR_054708,CM003006	W156C	The mutation does not affect the aggregation tendency of your protein.	The mutation does not affect the amyloid propensity of your protein	The mutation increases the chaperone binding tendency of your	The mutation reduces the protein stability

				protein.	
CM128259	G161D	The mutation does not affect the aggregation tendency of your protein.	The mutation does not affect the amyloid propensity of your protein.	The mutation does not affect the chaperone binding tendency of your protein.	The mutation severely reduces the protein stability.
CM1823494	F354S	The mutation decreases the aggregation tendency of your protein	The mutation decreases the amyloid propensity of your protein.	The mutation does not affect the chaperone binding tendency of your protein.	The mutation severely reduces the protein stability.
1067432,CM130686	W361R	The mutation does not affect the aggregation tendency of your protein.	The mutation does not affect the amyloid propensity of your protein.	The mutation does not affect the chaperone binding tendency of your protein.	The mutation severely reduces the protein stability.
CM1823503	W361S	The mutation does not affect the aggregation tendency of your protein.	The mutation does not affect the amyloid propensity of your protein	The mutation does not affect the chaperone binding tendency of your protein.	The mutation severely reduces the protein stability.

VAR_054735,CM003011	W649C	The mutation does not affect the aggregation tendency of your protein.	The mutation does not affect the amyloid propensity of your protein.	The mutation increases the chaperone binding tendency of your protein.	The mutation severely reduces the protein stability.

Supplementary table S6 overall results of HOPE analysis

**Rsid, HGMD, UniProt**	**mutations**	**hydrophobic property**	**size of the protein**	**location**	**Effects of mutation on the protein**
VAR_054707	I154R	The hydrophobicity of the wild-type and mutant residue differs and the mutation will cause loss of hydrophobic interactions in the core of the protein.	The size of both wild-type and mutant amino acids differ in size and the mutant residue is bigger than the wild-type residue.	The wild-type residue was buried in the core of the protein. The mutant residue is bigger and probably will not fit.	The variant is highly pathogenic with MetaRNN score of 0.99071544 and mutant residue is in highly conserved position
VAR_054735,CM003011	W649C	_	The size of both wild-type and mutant amino acids differ in size and the mutant residue is smaller than the wild-type residue	The mutation will cause an empty space in the core of the protein.	The variant is highly pathogenic with MetaRNN score of 0.99636817 and mutant residue is in highly conserved position

Supplementary table S7. Binding energy score achieved from the MMPBSA package of the GROMACS for 1st simulation run.

Simulations	Energy properties	Native protein	Mutant protein (I154R)
Run 1	van der Waal energy	-24.907 ± 24.936 kJ/mol	-35.479 ± 10.043 kJ/mol
	Electrostatic energy	-284.621 ± 24.759 kJ/mol	-170.760 ± 28.807 kJ/mol
	Polar solvation energy	283.932 ± 12.295 kJ/mol	198.875 ± 38.257 kJ/mol
	SASA energy	-11.942 ± 0.950 kJ/mol	-11.067 ± 1.158 kJ/mol
	Binding energy	-37.538 ± 10.409 kJ/mol	-18.431 ± 16.161 kJ/mol

Supplementary Figures

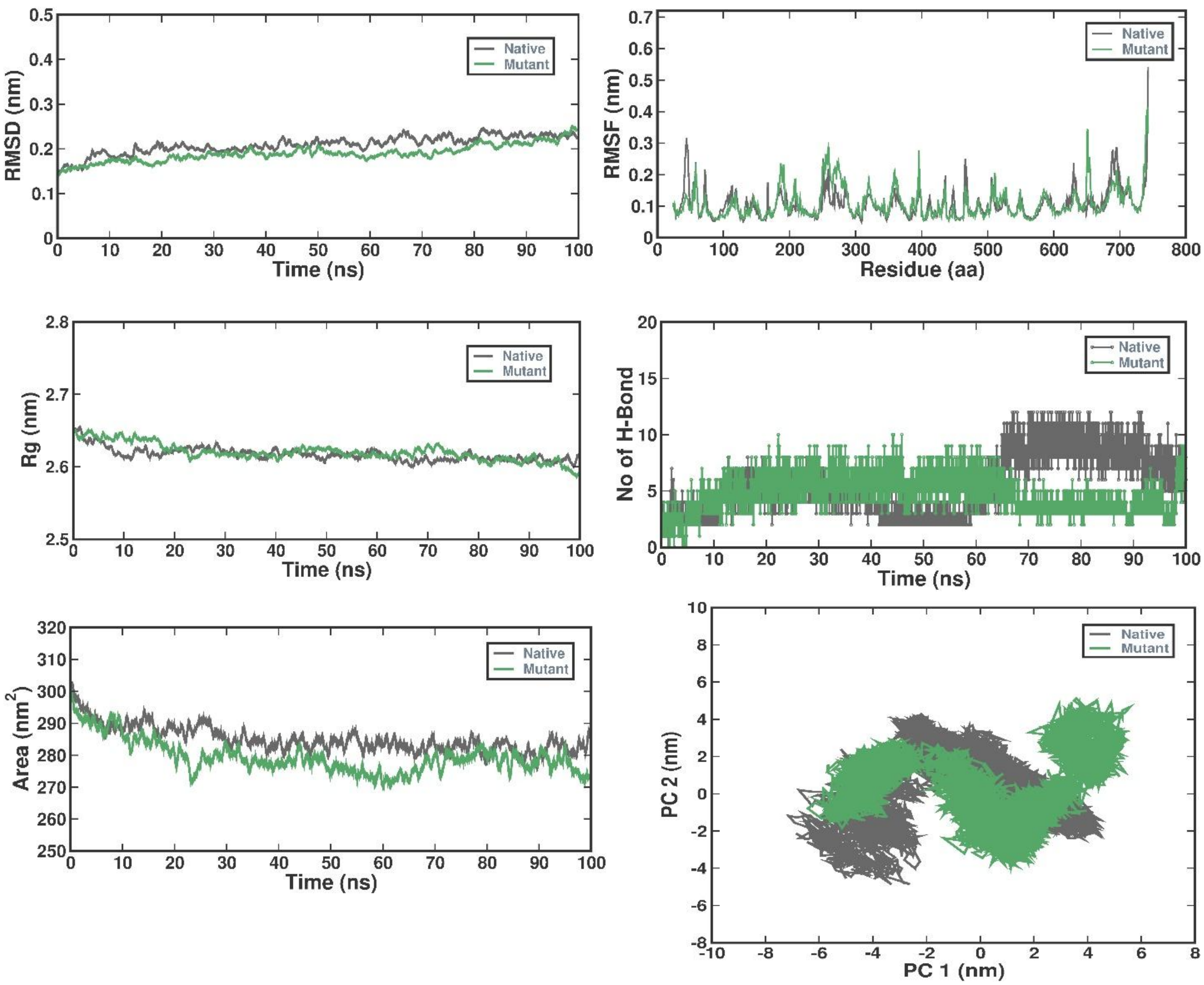

Supplementary figure S1. The Root Mean Square Deviation (RMSD) and The Root Mean square fluctuation (RMSF), H-bond and Radius of gyration for NAGLU native and I154R mutant was performed for 100ns time interval. Color schemes: Grey- Native and Green- I154R for 1st simulation run.

PRODRG-generated ligand topology

.itp file

```
[ moleculetype ]
; Name   nrexcl
1A9J    3
[ atoms ]
;  nr  type  resnr  resid  atom  cgnr  charge   mass
   1     H    1    1A9J    H13    1    0.415   1.0080
   2  OAlc    1    1A9J     O4    2   -0.655  15.9994
   3  CPos    1    1A9J     C8    3    0.233  12.0110
   4    HC    1    1A9J    H11    4    0.022   1.0080
   5    HC    1    1A9J    H12    5    0.060   1.0080
   6     C    1    1A9J     C6    6    0.080  12.0110
   7    HC    1    1A9J     H8    7    0.046   1.0080
   8    OE    1    1A9J     O2    8   -0.428  15.9994
   9  CPos    1    1A9J     C7    9    0.406  12.0110
  10    HC    1    1A9J     H9   10    0.040   1.0080
  11  OAlc    1    1A9J     O3   11   -0.672  15.9994
  12  HS14    1    1A9J    H10   12    0.457   1.0080
  13     C    1    1A9J     C3   13    0.251  12.0110
  14    HC    1    1A9J     H5   14    0.062   1.0080
  15     N    1    1A9J     N1   15   -0.757  14.0067
  16  HS14    1    1A9J     H4   16    0.368   1.0080
  17  CPos    1    1A9J     C2   17    0.793  12.0110
  18 OEOpt    1    1A9J     O1   18   -0.644  15.9994
  19     C    1    1A9J     C1   19   -0.523  12.0110
  20    HC    1    1A9J     H1   20    0.152   1.0080
  21    HC    1    1A9J     H2   21    0.152   1.0080
```

```
  22   HC    1   1A9J    H3   22    0.152   1.0080
  23  CPos   1   1A9J    C4   23    0.288  12.0110
  24   HC    1   1A9J    H6   24    0.019   1.0080
  25  OAlc   1   1A9J    O6   25   -0.645  15.9994
  26  HS14   1   1A9J   H15   26    0.460   1.0080
  27    C    1   1A9J    C5   27   -0.064  12.0110
  28   HC    1   1A9J    H7   28    0.090   1.0080
  29  OAlc   1   1A9J    O5   29   -0.578  15.9994
  30    H    1   1A9J   H14   30    0.420   1.0080
; total charge of the molecule:  -0.000
[ bonds ]
;  ai   aj  funct   c0         c1
    1    2    2   0.0972   1.9581e+07
    2    3    2   0.1430   8.1800e+06
    3    4    2   0.1090   1.2300e+07
    3    5    2   0.1090   1.2300e+07
    3    6    2   0.1530   7.1500e+06
    6    7    2   0.1100   1.2100e+07
    6    8    2   0.1430   8.1800e+06
    6   27    2   0.1530   7.1500e+06
    8    9    2   0.1430   8.1800e+06
    9   10    2   0.1090   1.2300e+07
    9   11    2   0.1400   8.5400e+06
    9   13    2   0.1540   4.0057e+06
   11   12    2   0.0971   7.9547e+06
   13   14    2   0.1090   1.2300e+07
   13   15    2   0.1450   5.2319e+06
   13   23    2   0.1560   3.0819e+06
```

```
   15   16    2   0.1010   2.1076e+07
   15   17    2   0.1360   1.0200e+07
   17   18    2   0.1230   1.6600e+07
   17   19    2   0.1520   5.4300e+06
   19   20    2   0.1090   1.2300e+07
   19   21    2   0.1090   1.2300e+07
   19   22    2   0.1090   1.2300e+07
   23   24    2   0.1090   1.2300e+07
   23   25    2   0.1430   8.1800e+06
   23   27    2   0.1560   3.0819e+06
   25   26    2   0.0972   1.9581e+07
   27   28    2   0.1100   1.2100e+07
   27   29    2   0.1420   3.2236e+06
   29   30    2   0.0972   1.9581e+07
[ pairs ]
;  ai   aj  funct  ;  all 1-4 pairs but the ones excluded in GROMOS itp
    1    4    1
    1    5    1
    1    6    1
    2    7    1
    2    8    1
    2   27    1
    3    9    1
    3   23    1
    3   28    1
    3   29    1
    4    7    1
    4    8    1
```

4	27	1
5	7	1
5	8	1
5	27	1
6	10	1
6	11	1
6	13	1
6	24	1
6	25	1
6	30	1
7	9	1
7	23	1
7	28	1
7	29	1
8	12	1
8	14	1
8	15	1
8	23	1
8	28	1
8	29	1
9	16	1
9	17	1
9	24	1
9	25	1
9	27	1
10	12	1
10	14	1
10	15	1

10	23	1
11	14	1
11	15	1
11	23	1
12	13	1
13	18	1
13	19	1
13	26	1
13	28	1
13	29	1
14	16	1
14	17	1
14	24	1
14	25	1
14	27	1
15	20	1
15	21	1
15	22	1
15	24	1
15	25	1
15	27	1
16	18	1
16	19	1
16	23	1
17	23	1
18	20	1
18	21	1
18	22	1

```
  23   30    1
  24   26    1
  24   28    1
  24   29    1
  25   28    1
  25   29    1
  26   27    1
  28   30    1
[ angles ]
;  ai    aj    ak  funct    angle      fc
    1     2     3     2    109.50    450.00
    2     3     4     2    111.40    532.00
    2     3     5     2    106.75    503.00
    2     3     6     2    111.00    530.00
    4     3     5     2    108.53    443.00
    4     3     6     2    109.50    448.00
    5     3     6     2    109.60    450.00
    3     6     7     2    108.00    465.00
    3     6     8     2    109.50    520.00
    3     6    27     2    111.00    530.00
    7     6     8     2    109.50    448.00
    7     6    27     2    108.53    443.00
    8     6    27     2    111.00    530.00
    6     8     9     2    119.00   2211.40
    8     9    10     2    108.53    443.00
    8     9    11     2    109.00   1680.51
    8     9    13     2    111.00    530.00
   10     9    11     2    111.00    530.00
```

10	9	13	2	111.00	530.00
11	9	13	2	109.50	520.00
9	11	12	2	109.50	450.00
9	13	14	2	106.75	503.00
9	13	15	2	115.00	610.00
9	13	23	2	111.00	530.00
14	13	15	2	108.53	443.00
14	13	23	2	107.60	507.00
15	13	23	2	109.50	520.00
13	15	16	2	115.00	460.00
13	15	17	2	130.00	3398.91
16	15	17	2	115.00	460.00
15	17	18	2	124.00	730.00
15	17	19	2	120.00	560.00
18	17	19	2	121.00	685.00
17	19	20	2	111.30	632.00
17	19	21	2	111.30	632.00
17	19	22	2	111.30	632.00
20	19	21	2	108.00	465.00
20	19	22	2	108.00	465.00
21	19	22	2	108.00	465.00
13	23	24	2	108.00	465.00
13	23	25	2	109.50	520.00
13	23	27	2	111.00	530.00
24	23	25	2	109.60	450.00
24	23	27	2	109.60	450.00
25	23	27	2	109.50	520.00
23	25	26	2	109.50	450.00

```
  6   27   23    2    109.50   520.00
  6   27   28    2    108.53   443.00
  6   27   29    2    109.50   520.00
 23   27   28    2    108.53   443.00
 23   27   29    2    109.50   520.00
 28   27   29    2    110.30   524.00
 27   29   30    2    109.50   450.00
[ dihedrals ]
; GROMOS improper dihedrals
;  ai   aj   ak   al  funct   angle     fc
   15   13   16   17    2      0.00   167.36
   17   15   18   19    2      0.00   167.36
[ dihedrals ]
;  ai   aj   ak   al  funct    ph0      cp     mult
    1    2    3    6    1      0.00     1.26    3
    2    3    6    8    1      0.00     5.92    3
    6    8    9   13    1    180.00     1.00    3
    8    6   27   23    1      0.00     5.92    3
    8    9   11   12    1      0.00     1.26    3
    8    9   13   15    1      0.00     5.92    3
   13   15   17   19    1    180.00    33.50    2
   13   23   25   26    1    180.00     1.00    3
   13   23   27   29    1    180.00     1.00    3
   15   13   23   27    1      0.00     5.92    3
   15   17   19   20    1    180.00     1.00    6
   23   13   15   17    1      0.00     3.77    6
   23   27   29   30    1      0.00     1.26    3
   27    6    8    9    1      0.00     1.26    3
```
